# Supplementary material for: A Novel Strategy to Fit and Validate Physiological Models: A Case Study of a Cardiorespiratory Model for Simulation of Incremental Aerobic Exercise
Source: Diagnostics (Basel). 2023 Feb 27;13(5):908. doi: 10.3390/diagnostics13050908 (PMC10000473; doi:10.3390/diagnostics13050908)
Supplement: Supplementary file 1 [file diagnostics-13-00908-s001.zip › diagnostics-2205168-supplementary.pdf]

TITLE

Supplementary Material for “A novel strategy to fit and validate physiological models: A case study of a cardiorespiratory model for simulation of incremental aerobic exercise”.

AUTHORS

Names

Carlos A. Sarmiento*, Leidy Y. Serna, Alher M. Hernández, Miguel Á. Mañanas

Correspondence Information

- Corresponding author: Carlos Andrés Sarmiento
- Address: Bioinstrumentation and Clinical Engineering Research Group, Bioengineering Department, Engineering Faculty,
- Universidad de Antioquia UdeA, Calle 70 # 52-51, Medellin 050016, Colombia.
- E-mail address: carlos.sarmiento@dea.edu.co

CARDIORESPIRATORY MODEL

All the involved subsystems and their principal components, outputs, and model interactions are presented in the schematic diagram shown in Figure S1. The used symbols are described in Table S1. A brief description of the model is presented below. Its development, nominal parameters values, and complete description of mechanisms are not reported hereon for brevity. The interested reader can refer to the previous paper for full details [1].

The italic style was implemented for the symbols related to the model parameters in order to differentiate them from model variables. Parameters are model constants (i.e., they do not change as a result of the model simulations), whereas variables are time-dependent signals.

Table S1. Symbols' description of the Model schematic block diagram

Symbol	Description
AT	Anaerobic threshold
BF	Breathing frequency
$\mathrm{CvCO_2}$	Carbon dioxide concentration in the venous blood
$\mathrm{CvO_2}$	Oxygen concentration in the venous blood
$\mathrm{E_{max,jv}}$	Regulated end-systolic elastance of each ventricle
$FiCO_2$	Inspired fractions of dry carbon dioxide
FiO_2	Inspired fractions of dry oxygen
HR	Heart rate
I	The action of central command
MRV	Metabolically related neural drive component to the ventilation
Nd	Neural drive signal
Pao	The pressure at the airway opening
P_{atm}	Atmospheric pressure
$\mathrm{PbCO_2}$	Brain carbon dioxide partial pressure
$\mathrm{P_{sa}}$	Systemic arterial pressure signal
$\mathrm{Q_{jp}}$	Blood flow of each peripheral compartment
$\mathrm{Q_{pp}}$	Blood flow from the pulmonary peripheral compartment
$\mathrm{R_{jp}}$	Regulated resistance of each peripheral compartment
T_C	Overall duration of the muscular contraction
T_{im}	Time duration of the muscular contraction-relaxation cycle
$\dot{\mathrm{V}}$	Airflow
$\dot{\mathrm{V}}\mathrm{E}$	Total minute ventilation
$\dot{V}CO_2$	Carbon dioxide output
$\dot{V}O_2$	Oxygen uptake
$\mathrm{V_{u,jv}}$	Regulated unstressed volume of each venous compartment
VT	Tidal volume
TI	Inspiratory time

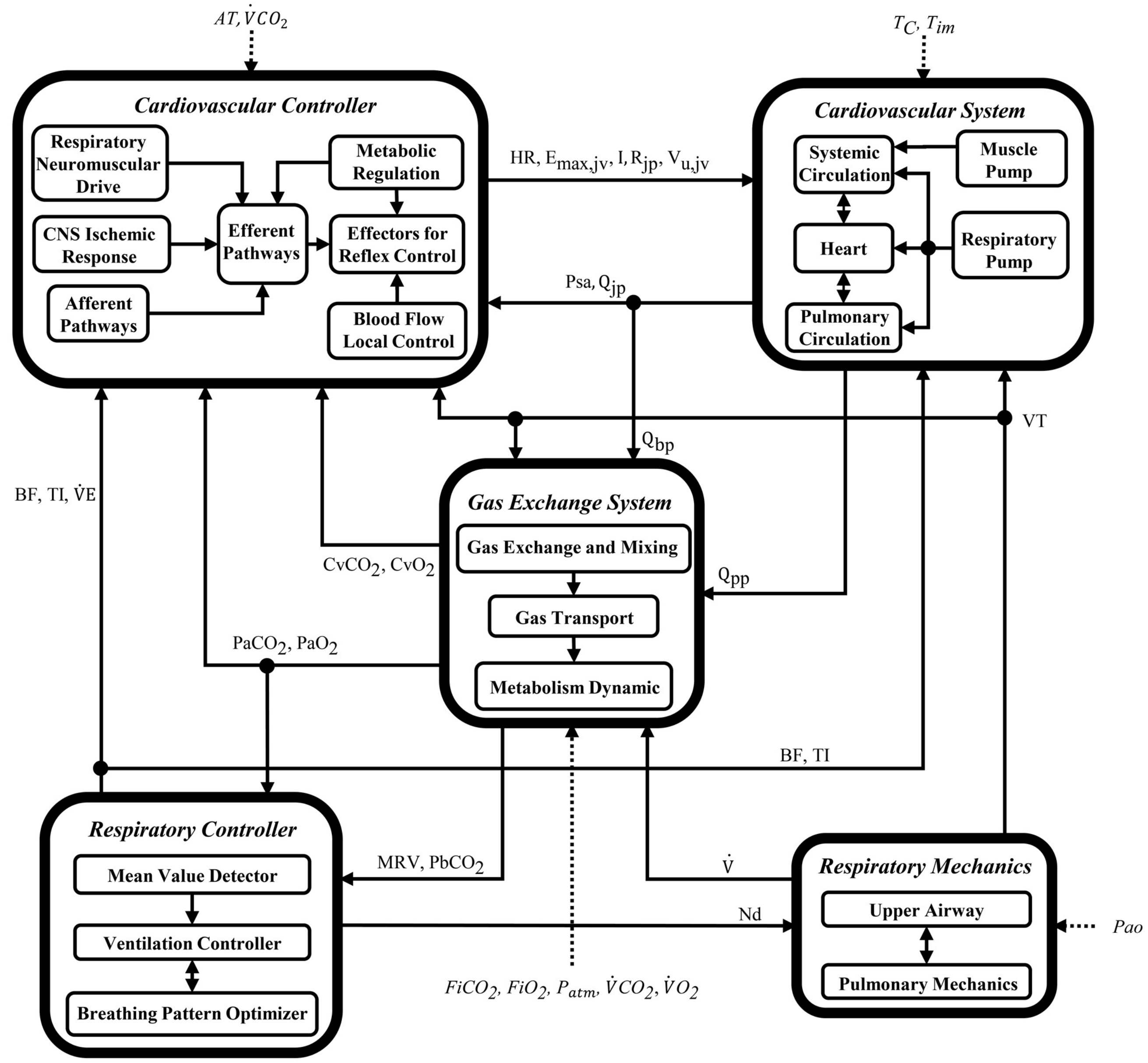

Figure S1. Schematic diagram of the model under study. The dashed lines denote model inputs. See Table S1 for symbols description

EXPERIMENTAL DATA

The experimental data used correspond to those recorded to build and evaluate the cardiorespiratory model [1].

All the procedures were approved by the Human Research Ethical Committee of the Department of University Research (SIU) of the University of Antioquia (approval certificate17-59-711) and are conformed to the Declaration of Helsinki. All volunteers' written consents were obtained after giving them the complete information of the experimental protocol and the possible risks.

Thirty Hispanic adult male volunteers performed the experimental test (28.3 ± 6.9 years old, 173.6 ± 6.0 cm height, 76.5 ± 9.3 kg weight). They were considered healthy, not overweight, with physical fitness that included sedentary subjects and subjects accustomed to physical training, non-smokers, no history, or symptoms of cardiovascular, pulmonary, metabolic, or neurological diseases, and without pacemakers or other implanted electrical stimulators.

The volunteers performed an incremental, submaximal, and multistate cardiopulmonary exercise test in a programmable cycle ergometer under controlled environment conditions (26.6 ± 1.7ºC, 64.0 ± 5.1% relative humidity, and 640 mmHg). The test lasted 45 minutes, divided into five stages: rest (2 minutes), warm-up (4 minutes), exercise (25 minutes), recovery (9 minutes), and final rest (5 minutes). Five incremental steps of 25W of load were evaluated during the exercise stage, with a duration of 5 minutes each and at a constant pedaling speed of 60 RPM.

The records comprise signals of $\dot{V}O_2$, $\dot{V}CO_2$, $\dot{V}E$, TI, VT, BF, HR, PAO_2 and $PACO_2$; systolic (PS), diastolic (PD) and mean (PM) arterial blood pressures; and environment and subjects' features such as P_{atm}, $FiCO_2$, FiO_2, AT and VTn.

MODEL EQUATIONS AND PARAMETERS

A quantitative description of the model equations mainly related to the fitting process is presented in the following section. Full details of each of the systems and controllers' equations, and the nominal values of the parameters can be consulted in the previous work [1].

Cardiovascular System

The main elements of the equations presented include pressures (P), inertances (L), blood flows (Q), hydraulic resistances (R) and blood volumes (V). Different subscripts are used to indicate a specific compartment: sa, systemic arteries; lv, left ventricle; s, splanchnic vascular beds; am, active skeletal muscle vascular beds. The systemic circulation describes peripheral and venous circulation (subscripts p and v) in each compartment. $\mathrm{P_{abd}}$, $\mathrm{P_{im}}$ and $\mathrm{P_{thor}}$ are the abdominal pressure, the extravascular pressure of the active muscle veins (i.e., the intramuscular pressure) and the intrathoracic pressure, respectively. The subscripts max, min, T, and u are used to indicate maximum, minimum, total and unstressed values, respectively.

Systemic Arteries

$$L_{sa} \cdot \frac{\mathrm{dQ_{sa}}}{\mathrm{dt}} = (\mathrm{P_{sa}} - \mathrm{P_{thor}}) - R_{sa} \cdot \mathrm{Q_{sa}} - \mathrm{P_{sp}} \quad \text{(S1)}$$

Systemic Peripheral and Venous Circulation

$$\mathrm{R_{amv}} = k_{r,am}/\mathrm{V_{Tamv}} \quad \mathrm{I} > 0 \quad \text{(S2)}$$

$$\mathrm{P_{amv}} = P_0 \cdot \left[1 - \left(\frac{\mathrm{V_{Tamv}}}{\mathrm{V_{u,amv}}}\right)^{-3/2}\right] + \mathrm{P_{im}} \quad \mathrm{V_{Tamv}} < \mathrm{V_{u,amv}} \quad \text{(S3)}$$

Where P_0 is a constant parameter.

The Heart

$$\mathrm{R_{lv}} = KR_{lv} \cdot \mathrm{P_{max,lv}} \quad \text{(S4)}$$

$$\mathrm{P_{max,lv}(t)} = \varphi(\mathrm{t}) \cdot \mathrm{E_{max,lv}} \cdot (\mathrm{V_{lv}} - V_{u,lv}) + [1 - \varphi(\mathrm{t})] \cdot P_{0,lv} \cdot \left(\exp^{(K_{E,lv} \cdot \mathrm{V_{lv}})} - 1\right) \quad 0 \le \varphi(\mathrm{t}) \le 1 \quad \text{(S5)}$$

Where $\varphi(\mathrm{t})$ is the ventricle activation function, with $\varphi(\mathrm{t}) = 1$ at maximum contraction and $\varphi(\mathrm{t}) = 0$ at complete relaxation; $\mathrm{E_{max,lv}}$ is the left ventricular elastance at the instant of maximum contraction; $P_{0,lv}$ is a parameter that describe the end-diastolic pressure-volume relationship in the left ventricle.

Respiratory Pump

$$\mathrm{P_{jmax}} = P_{jmax,n} + g_j \cdot \Delta\mathrm{VT} \quad \text{(S6)}$$

$$\mathrm{P_{jmin}} = P_{jmin,n} - g_j \cdot \Delta\mathrm{VT} \quad \text{(S7)}$$

Where the subscript j indicates the compartment abdominal (abd) and thoracic (thor); the subscript n denotes nominal values.

Muscle Pump

$$\mathrm{P_{im}} = A_{im} \cdot \psi(\mathrm{t}) \quad \text{(S8)}$$

Where $\psi(\mathrm{t})$ is the activation function of skeletal muscle fibers (with $\psi(t) = 1$ at maximum contraction, $\psi(t) = 0$ at complete relaxation).

Cardiovascular Controller

Blood Flow Local Control

$$\mathrm{R_{amp}} = \frac{\mathrm{R_{amp,n}}}{\left(1 + \mathrm{x_{am,O_2}} + \mathrm{x_{met}}\right)} \quad \text{(S9)}$$

$$\tau_{O_2} \cdot \frac{\mathrm{dx_{am,O_2}}}{\mathrm{dt}} = -\mathrm{x_{am,O_2}} - g_{am,O_2} \cdot \left(\mathrm{C_{vam,O_2}} - C_{vam,O_2n}\right) \quad \text{(S10)}$$

$$\mathrm{C_{vam,O_2}} = \mathrm{C_{aO_2}} - \frac{\mathrm{M_{O_2,amp}}}{\mathrm{Q_{amp}}} \quad \text{(S11)}$$

$$\mathrm{M_{O_2,amp}} = M_{O_2,ampn} \cdot (1 + \mathrm{x_M}) \quad \text{(S12)}$$

$$\tau_M \cdot \frac{\mathrm{dx_M}}{\mathrm{dt}} = -\mathrm{x_M} + g_M \cdot \mathrm{I} \quad \text{(S13)}$$

$$\tau_{met} \cdot \frac{\mathrm{dx_{met}}}{\mathrm{dt}} = -\mathrm{x_{met}} + \Phi_{\mathrm{met}}(\mathrm{t} - D_{met}) \quad \text{(S14)}$$

$$\Phi_{\mathrm{met}}(\mathrm{t}) = \frac{\Phi_{min} + \Phi_{max} \cdot \exp^{\left(\frac{\mathrm{I}-I_{0,met}}{k_{met}}\right)}}{1 + \exp^{\left(\frac{\mathrm{I}-I_{0,met}}{k_{met}}\right)}} \tag{S15}$$

Where x_{am,O_2} and x_{met} are state variables, representing the effect of tissue hypoxia and of the other vasodilatory substances; τ_{O_2}, τ_{met} and τ_M are time constants; g_{am,O_2} is a constant gain factor; C_{vam,O_2} and C_{vam,O_2n} represents oxygen venous concentration in the active muscle and its nominal value, respectively; C_{aO_2} denotes the oxygen arterial concentration; $M_{O_2,amp}$ and $M_{O_2,ampn}$ are the oxygen consumption rate in the active muscle compartment and their nominal value, respectively; x_M is a state variable; $\Phi_{met}(t)$ is the output of the sigmoidal function; D_{met} is a constant delay; Φ_{min} is the lower saturation of the static sigmoidal characteristic; $I_{0,met}$ is the value of I at the central point of the sigmoid; k_{met} is a parameter related to the slope of the sigmoid.

Efferent Pathways

$$\mathrm{f_{sj}} = \begin{cases} f_{es,\infty} + \left(f_{es,0} - f_{es,\infty}\right) \cdot \exp^{(k_{es} \cdot \mathrm{f_{asj}})} + \gamma_{\mathrm{sj}}(\mathrm{I}) & \mathrm{f_{sj}} < f_{es,max} \\ f_{es,max} & \mathrm{f_{sj}} \geq f_{es,max} \end{cases} \tag{S16}$$

$$\mathrm{f_{asj}} = W_{t,sj} \cdot \mathrm{Nt} + W_{b,sj} \cdot \mathrm{f_{ab}} + W_{c,sj} \cdot \mathrm{f_{ac}} + W_{p,sj} \cdot \mathrm{f_{ap}} - \theta_{\mathrm{sj}} \tag{S17}$$

$$\mathrm{f_v} = \frac{f_{ev,0} + f_{ev,\infty} \cdot \exp^{\left(\frac{\mathrm{f_{ab}} - f_{ab,0}}{k_{ev}}\right)}}{1 + \exp^{\left(\frac{\mathrm{f_{ab}} - f_{ab,0}}{k_{ev}}\right)}} - W_{t,v} \cdot \mathrm{Nt} + W_{c,v} \cdot \mathrm{f_{ac}} + W_{p,v} \cdot \mathrm{f_{ap}} - \theta_v + \gamma_{\mathrm{v}}(\mathrm{I}) \tag{S18}$$

$$\gamma_{\mathrm{i}} = \frac{\gamma_{i,min} + \gamma_{i,max} \cdot \exp^{\left(\frac{\mathrm{I}-I_{0,i}}{k_{cc,i}}\right)}}{1 + \exp^{\left(\frac{\mathrm{I}-I_{0,i}}{k_{cc,i}}\right)}} \tag{S19}$$

Where the subscript j=h, p, v indicates sympathetic activity to heart, peripheral resistances, and veins, respectively; $f_{es,max}$, $f_{es,0}$, $f_{ev,0}$, $f_{es,\infty}$, $f_{ev,\infty}$, $f_{ab,0}$, k_{es}, and k_{ev} are constant parameters; f_{sj} is the sympathetic activity; f_v is the frequency of spike in the efferent vagal fibers; f_{asj} is the weighted sum of Nt, the offset term caused by the CNS hypoxia(θ_{sj}), the afferent activities from baroreceptors (f_{ab}), lung stretch receptors (f_{ap}) and chemoreceptors (f_{ac}); $W_{b,sj}$, $W_{c,sj}$ and $W_{p,sj}$ are weighting factors that relate f_{ab}, f_{ac} and f_{ap} with f_{asj}; $W_{c,v}$ and $W_{p,v}$ are weighting factors that relate f_{ac} and f_{ap} with f_v; θ_v is an offset term; of a characteristic sigmoid function; the subscript i=sp, sv and sh indicate sympathetic activity to peripheral arteries, veins and heart, and v denotes parasympathetic activity; $\gamma_{i,min}$ denotes the lower saturation values of sigmoid functions; I_0 and k_{cc} are constant parameters values related to the central point and the slope of characteristic sigmoid functions, respectively.

Effectors for Reflex Control

$$\frac{1}{\mathrm{HR}} = \Delta\mathrm{T_v} + \Delta\mathrm{T_s} + T_0 \tag{S20}$$

$$\tau_{T,v} \cdot \frac{\mathrm{d}\Delta\mathrm{T_v}(\mathrm{t})}{\mathrm{dt}} = -\Delta\mathrm{T_v}(\mathrm{t}) + \sigma_{\mathrm{T,v}}(\mathrm{t}) \tag{S21}$$

$$\sigma_{\mathrm{T,v}}(\mathrm{t}) = G_{T,v} \cdot \mathrm{f_v}\left(\mathrm{t} - D_{T,v}\right) \tag{S22}$$

Where ΔT_v represents the changes in heart period induced by parasympathetic stimulation; ΔT_s represents the changes in heart period induced by sympathetic stimulation; $\sigma_{T,v}$ is the output of characteristic function related to parasympathetic activity; $D_{T,v}$ is a constant delay of the mechanism; $\tau_{T,v}$ is a time constant.

Respiratory Mechanics

The main elements of the equations related to the Respiratory Mechanics include compliances (C), elastances (E), pressures (P), resistances (R), volumes (V) and flows ($\dot{V}$).

$$\dot{\mathrm{V}}(\mathrm{t}) = \frac{\mathrm{G_{AW}}}{R_{rs}} \cdot \left((\mathrm{P_{musc}}(\mathrm{t}) - Pao) - E_{rs} \cdot \mathrm{V}(\mathrm{t})\right) \tag{S23}$$

Where R_{rs} is the overal ressitence of the ventilatory system; G_{AW} is the airway flow gain factor; Pao is the airway pressure.

Respiratory Controller

Ventilation Controller

$$\dot{\mathrm{V}}\mathrm{E} = \dot{\mathrm{V}}\mathrm{A} + \mathrm{BF} \cdot \mathrm{VD} \tag{S24}$$

$$\mathrm{VD} = GV_{dead} \cdot \dot{\mathrm{V}}\mathrm{A} + V0_{dead} \tag{S25}$$

$$\dot{\mathrm{V}}\mathrm{A} = \dot{V}A_{rest} \cdot (KpCO_2 \cdot \mathrm{PamCO_2} + KcCO_2 \cdot \mathrm{PmbCO_2} + \mathrm{G_3} + KcMRV \cdot \mathrm{MRV} - Kbg) \tag{S26}$$

$$\mathrm{G_3} = \begin{cases} KpO_2 \cdot (104 - \mathrm{PamO_2})^{4.9} & \mathrm{PamO_2} < 104 \\ 0 & \mathrm{PamO_2} \geq 104 \end{cases} \tag{S27}$$

Where $\dot{V}A$ is the alveolar ventilation; $\dot{V}A_{rest}$ is the basal value of alveolar ventilation; VD is the dead space volume; $PamO_2$, $PamCO_2$ and $PbmCO_2$ are the mean values of PaO_2, $PaCO_2$ and $PbCO_2$ in each respiratory cycle.

Breathing Pattern Optimizer

$$\dot{W}_T = \dot{W}_I + \lambda_2 \cdot \dot{W}_E \tag{S28}$$

$$\dot{W}_I = \frac{1}{(t_1 + t_2)} \int_0^{t1} \left[\frac{P_{musc}(t)}{\xi_1^n \xi_2^n} + \lambda_1 \cdot \ddot{V}(t)^2 \right] dt \tag{S29}$$

$$\xi_1 = 1 - P_{musc}(t)/P_{max} \tag{S30}$$

Where ξ_1 and ξ_2 are efficiency factors.

Gas Exchange System

Gas Exchange and Mixing

$$V_{gas} \cdot \frac{dPA_{gas}}{dt} = \begin{cases} 863 \cdot (Q_{pp})(C_{v,gas} - C_{a,gas}) + \dot{V} \cdot (P_{d(5)gas} - PA_{gas}) & \dot{V}(t) \geq 0 \\ 863 \cdot (Q_{pp})(C_{v,gas} - C_{a,gas}) & \dot{V}(t) < 0 \end{cases} \tag{S31}$$

$$C_{aO_2} = (C_1 \cdot Z) \cdot \frac{{F_{O_2}}^{1/a_1}}{1 + {F_{O_2}}^{1/a_1}} \tag{S32}$$

$$V_{gas} = V(t) + VL_{gas} \tag{S33}$$

Where the subscripts gas represent the specific gas (O_2 or CO_2); V denotes pulmonary volume; Q_{pp} is the peripheral pulmonary blood flow; $C_{v,gas}$ and $C_{a,gas}$ denote venous and arterial gas concentrations, respectively; $P_{d(5)gas}$ denotes the gas pressure in the fifth dead space compartment; Z is the molar conversion factor from mmol/l to l/l; F_{O_2}denotes the fraction of O_2; a_1 is a parameter associated with the Bohr and Haldane effects regarding the O_2 dissociation in blood.

Gas transport

$$SbCO_2 \cdot \frac{dPvbCO_2}{dt} = MRBCO_2 + Q_{bp} \cdot SCO_2(PaCO_2 - PvbCO_2) - h \tag{S34}$$

Where $SbCO_2$ is the dissociation slope for CO_2 in the brain tissue; Q_{bp} is Blood flow of brain peripheral compartment; SCO_2 is the dissociation slope for CO_2 in the blood; h is a cerebral blood flow constant.

REFERENCES

[1] C.A. Sarmiento, A.M. Hernandez, L.Y. Serna Higuita, M.Á. Mañanas, An integrated mathematical model of the cardiovascular and respiratory response to exercise: Model-building and comparison with reported models, Am. J. Physiol. Circ. Physiol. (2021) ajpheart.00074.2020. https://doi.org/10.1152/ajpheart.00074.2020.
